# Impact of Drug-Mediated Inhibition of Intestinal Transporters on Nutrient and Endogenous Substrate Disposition…an Afterthought?

**DOI:** 10.3390/pharmaceutics16040447

**Published:** 2024-03-24

**Authors:** Kshitee Kharve, Andrew S. Engley, Mary F. Paine, Jason A. Sprowl

**Affiliations:** 1 Department of Pharmaceutical Sciences, School of Pharmacy and Pharmaceutical Sciences, University at Buffalo, State University of New York, Buffalo, NY 14214, USA; kshitees@buffalo.edu; 2 Department of Pharmaceutical Sciences, College of Pharmacy and Pharmaceutical Sciences, Washington State University, Spokane, WA 99202, USA; andrew.engley@wsu.edu (A.S.E.); mary.paine@wsu.edu (M.F.P.)

**Keywords:** absorption, ASBT, endogenous, interactions, intestine, MCT1, nutrients, NPC1L1, OSTα/β, PCFT, SGLT1, THTR2, transporters

## Abstract

A large percentage (~60%) of prescription drugs and new molecular entities are designed for oral delivery, which requires passage through a semi-impervious membrane bilayer in the gastrointestinal wall. Passage through this bilayer can be dependent on membrane transporters that regulate the absorption of nutrients or endogenous substrates. Several investigations have provided links between nutrient, endogenous substrate, or drug absorption and the activity of certain membrane transporters. This knowledge has been key in the development of new therapeutics that can alleviate various symptoms of select diseases, such as cholestasis and diabetes. Despite this progress, recent studies revealed potential clinical dangers of unintended altered nutrient or endogenous substrate disposition due to the drug-mediated disruption of intestinal transport activity. This review outlines reports of glucose, folate, thiamine, lactate, and bile acid (re)absorption changes and consequent adverse events as examples. Finally, the need to comprehensively expand research on intestinal transporter-mediated drug interactions to avoid the unwanted disruption of homeostasis and diminish therapeutic adverse events is highlighted.

## 1. Introduction

A semi-impervious membrane bilayer is necessary to protect intracellular components and whole organisms from potentially harmful xenobiotics [1]. Conversely, cellular homeostasis requires nutrients and endogenous substrates to pass through this barrier. Membrane transporters are key regulators of this process and are critical for the absorption of essential biomolecules and therapeutics. As of 2018, 62% of FDA-approved drugs were designed for oral administration, and new drugs continue to be developed for delivery through this non-invasive route [2]. As with nutrients and endogenous substrates, systemic exposure to drugs requires passage through enterocytes (and hepatocytes).

Numerous membrane transporters within enterocytes have been identified and characterized regarding the regulation of nutrient, endogenous substrate, or drug (re)absorption [3,4], and many have been recognized by the International Transporter Consortium (ITC) as important to consider throughout drug development [5]. These transporters include the efflux transporters P-glycoprotein (P-gp, encoded by the *ABCB1* gene), breast cancer resistance protein (BCRP, encoded by the *ABCG2* gene), and multidrug resistance proteins 2 and 3 (MRP2 and MRP3, encoded by the *ABCC2* and *ABCC3* genes, respectively), as well as the uptake transporters organic anion transporting polypeptide 2B1 (OATP2B1, encoded by the *SLCO2B1* gene), peptide transporters 1 and 2 (PEPT1 and PEPT2, encoded by the *SLC15A1* and *SLC15A2* genes, respectively), monocarboxylate transporter 1 (MCT1, encoded by the *SLC16A1* gene), apical sodium dependent bile acid transporter (ASBT, encoded by the *SLC10A2* gene), organic solute transporters α and β (OSTα and OSTβ, encoded by the *SLC51A* and *SLC51B* genes, respectively), and thiamine transporters 1 and 2 (THTR1 and THTR2, encoded by the *SLC19A2* and *SLC19A3* genes, respectively). In addition to these ITC-recognized transporters, sodium glucose transporter 1 (SGLT1, encoded by the *SLC5A1* gene) has been subject to many investigations due to its role in mediating intestinal glucose uptake and sensitivity to certain drugs, along with the proton-coupled folate transporter (PCFT, encoded by the *SLC46A1* gene) and Niemann-Pick C1-Like1 transporter (NPC1L1, encoded by the *SLC65A2* gene) due to their involvement in folate and cholesterol absorption, respectively.

The roles of many of the above transporters within the intestine, including P-gp, MRPs, PEPT1, and OATP2B1, are detailed in comprehensive reviews [6,7]; however, previous studies involving reduced-activity genetic variants, drug–drug interactions, or food–drug interactions have arguably been predicated upon investigating their contributions to xenobiotic disposition. Consequently, an insufficient consideration of drugs that alter the endogenous function of intestinal transporters, which contributes to unwanted effects such as nutrient malabsorption, can impact drug development and therapeutic outcomes. For example, although not a transporter expressed within enterocytes, the activity of the hepatic bile salt export pump (BSEP, encoded by the *ABCB11* gene) can be inhibited by several tyrosine kinase inhibitors (TKIs), a class of drugs designed to treat various forms of cancer. These drugs can potentially disrupt BSEP-mediated bile acid secretion and promote cholestasis through the hepatic accumulation of bile acids [8]. Some TKIs have recently been shown to inhibit the activity of a variety of other transporters in a non-competitive manner [9,10]. Moreover, many drugs are associated with adverse events that can be partially explained by nutrient deficiencies. For example, patients treated with some TKIs have experienced symptoms that include hypoglycemia or thiamine deficiency. In fact, the symptoms of thiamine deficiency were a major challenge associated with the regulatory approval of fedratinib (detailed below).

Due to the above-highlighted challenges, this review underscores the importance of understanding how intestinal transporters are impacted by therapeutics and the consequent effects on nutrient or endogenous substrate disposition or homeostasis. Examples of altered nutrient or endogenous substrate disposition following exposure to xenobiotics, as well as the outcomes and characterization of these events, are described. Opportunities to improve the prediction and characterization of transporter-mediated drug interactions are also provided.

## 2. Examples of Altered Nutrient and Endogenous Substrate Disposition due to Drugs

Reports of drugs that may alter nutrient or endogenous substrate disposition, as well as the consequent biological outcomes, are limited. Indeed, current clinically relevant observations are largely limited to drugs that impact substrate interactions with SGLT1, THTR2, PCFT, MCT1, NPC1L1, ASBT, or OSTα/β. The expression, protein abundance, and membrane localization of these transporters are presented (Table 1), while details of altered substrate disposition in the presence of certain drugs are outlined below.

## 3. Disruption of Glucose Disposition

**Figure 1:**
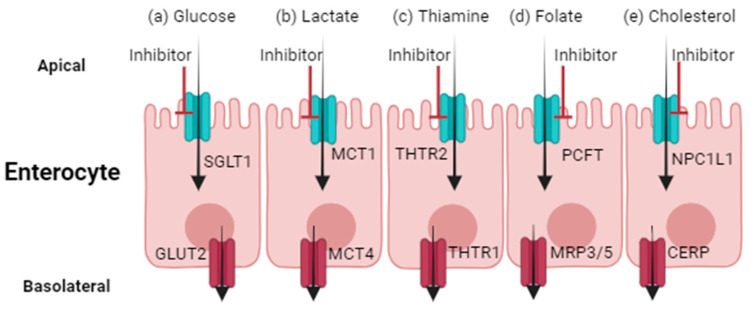
Transporter-mediated drug-nutrient or drug-endogenous substrate interactions within enterocytes. Reduced activity of SGLT1 (**a**), MCT1 (**b**), THTR2 (**c**), PCFT (**d**), or NPC1L1 (**e**) at the apical membrane can result in reduced absorption of glucose, lactate, thiamine, folate, and cholesterol, respectively. GLUT2, MCT4, THTR1, MRP3 or 5, and CERP mediate basolateral efflux of glucose, lactate, thiamine, folate, or cholesterol, respectively. SGLT1, sodium-glucose linked transporter 1. GLUT2, glucose transporter 2. MCT1/4, monocarboxylic acid transporter 1 or 4. THTR1/2, thiamine transporter 1 or 2. PCFT, proton-coupled folate transporter. MRP3/5, multidrug resistance protein 3 or 5. NPC1L1, Niemann-Pick C1-Like1 transporter. CERP, cholesterol efflux regulatory protein. Created using BioRender.

The movement of glucose, the primary source of mammalian cell energy, through the apical membrane of enterocytes is highly dependent on SGLT1. SGLT1 is a 73 kDa protein that is responsible for intestinal glucose uptake using symport via a sodium gradient, after which glucose moves into the portal circulation through glucose transporter 2 (GLUT2, encoded by the *SLC2A2* gene) at the basolateral membrane (Figure 1). SGLT1 is also expressed at the apical membrane in renal proximal tubule cells, where it contributes to glucose reabsorption into the systemic circulation.

The importance of SGLT1 is best represented by individuals with genetic loss-of-function variants who suffer from glucose–galactose malabsorption, diarrhea, and hypoglycemia resulting from diminished glucose uptake into enterocytes [19]. Consistent with these observations, *Sglt1*-deficient rodents were reported to exhibit these symptoms [20]. Such a disrupted glucose disposition was critical to the development of gliflozins, which are selective inhibitors of SGLT2, the major mediator of renal glucose reabsorption, which is used to treat diabetes. The gliflozins vary in SGLT1 inhibitory potency, especially sotagliflozin (Table 2), which is a dual SGLT1/2 inhibitor that reduces postprandial and fasting blood glucose in patients with types 1 or 2 diabetes [21,22]. The simultaneous inhibition of Sglt1 and Sglt2 in rodents was considered to be due to a significantly greater reduction in renal glucose reabsorption compared to the loss of Sglt2 activity alone [23]. A dose-limiting adverse event associated with sotagliflozin is diarrhea, which results from carbohydrate accumulation in the gastrointestinal tract, along with a reversed osmotic flow of water following the loss of SGLT1 activity within enterocytes [24].

Beyond the gliflozins, other drugs have been linked to the inhibition of glucose transport. The TKIs lapatinib and erlotinib, which are used to treat breast and non-small cell lung cancers, respectively, have been shown to diminish glucose uptake in vitro; erlotinib-resistant cells are impervious to this event [25,26,27]. Whether this event is mediated predominantly through SGLTs or other glucose transporters requires further investigation. Studies should extend to TKIs other than lapatinib and erlotinib that have been linked to clinical hypoglycemia and diarrhea, where the mechanisms responsible remain poorly understood [28]. For example, reduced blood glucose concentrations and dependency on anti-diabetic medications have been reported in patients treated with sorafenib, dasatinib, sunitinib, and imatinib (Table 2) [28]. Several hypotheses to explain these observations have evolved, which include regression of pancreatic islet cells, IGF-1 regulation via HIF1-α, and NF-kB activation. Considering the mechanisms by which TKIs impact other transporters [9,47], and that TKI-induced hypoglycemia and diarrhea phenotypes are consistent with a loss of intestinal glucose uptake, further research is needed to understand the effects of TKIs on SGLT1 function.

## 4. Disruption of Thiamine Absorption

Thiamine, also known as vitamin B1, is an essential water-soluble nutrient obtained through dietary consumption. Thiamine absorption into enterocytes is largely mediated by THTR2, a 56 kDa protein located at the apical membrane of enterocytes (Figure 1). The importance of THTR2 in this process is best characterized by reduced intestinal absorption and subsequent reduced systemic concentrations of thiamine in *Thtr2*-deficient mice [48]. Following uptake into enterocytes, THTR1 mediates the movement of thiamine through the basolateral membrane into the portal vein and eventually systemic circulation. THTR1 and THTR2, which are ubiquitously expressed, also mediate thiamine uptake from the circulation into cells, along with its reabsorption from renal proximal tubule cells [49]. Thiamine (and small quantities of metabolites) is largely excreted into the urine by glomerular filtration and tubular secretion, while hepatic uptake of thiamine occurs via organic cation transporter 1 (OCT1, encoded by the *SLC22A1* gene).

Patients with *THTR2* reduced-activity variants are at increased risk for Wernicke’s encephalopathy, a severe neurological condition caused by prolonged thiamine deficiency through malnutrition or malabsorption [50]. Although inhibitors were not designed to clinically target THTR2, the transporter was recently shown to be sensitive to inhibition by various drugs (Table 2). The first indication of this event occurred following the termination of Phase 3 clinical trials of the TKI fedratinib when many patients developed Wernicke’s encephalopathy. Follow-up in vitro studies revealed that fedratinib is a THTR2 substrate and inhibitor at low, clinically relevant concentrations (Table 2), which would diminish thiamine absorption through enterocytes and promote Wernicke’s encephalopathy [29]. Since this discovery, the occurrence of Wernicke’s encephalopathy has been decreased by monitoring thiamine concentrations before and during treatment. However, other drugs, including amitriptyline and hydroxychloroquine, have been identified as THTR inhibitors using in vitro models (Table 2) [30,31]. Future clinical interaction studies associated with these drugs and thiamine disposition changes are highly recommended; transporters beyond THTR2 should be also considered. Evidence for this expansion involves recent clinical and animal data showing an unexpected increase in systemic thiamine concentrations upon the administration of trimethoprim, an antifolate antibiotic with THTR2-inhibitory properties [32]. This increase in systemic thiamine concentrations was believed to result from the simultaneous inhibition of thiamine hepatic uptake and its clearance via OCT1 by trimethoprim. Therefore, future investigations of potential THTR2 inhibitors, such as metformin and verapamil [14], and their ability to alter systemic thiamine concentrations should include assessments of other transporters that contribute to the balance between thiamine absorption and clearance, including THTR2 and OCT1. Additionally, changes to thiamine metabolism may need to be considered.

## 5. Disruption of Folate Disposition

Folate, or vitamin B-9, is essential for DNA synthesis and cell growth. Folate is obtained from the diet via absorption into the apical membrane of enterocytes by PCFT, a 50 kDa protein that uses the symport of protons to drive transport (Figure 1). Cellular folate exits the basolateral membrane of enterocytes into the portal vein via a currently unconfirmed mechanism that is believed to involve MRPs [15]. The uptake of folate from the circulation into cells then occurs by PCFT, as well as the reduced folate carrier (RFC, encoded by the *SLC19A1* gene) and folate receptors. The importance of PCFT is highlighted by genetically deficient mice that develop severe anemia and pancytopenia resulting from systemic folate deficiency, which is largely due to disrupted intestinal folate uptake [51]. Consistent with these phenotypes, patients with reduced-activity *PCFT* variants have impaired intestinal folate absorption, along with impaired transport into the central nervous system, which collectively leads to anemia and, in many patients, seizures or mental deficiencies [52].

Based on the importance of folate and PCFT, no drugs have been designed with the goal of inhibiting PCFT; however, antifolates, including methotrexate and raltitrexed, are PCFT substrates [53]. Oral methotrexate is associated with adverse events that include gastrointestinal toxicity, anemia, and myelosuppression, each of which can be attributed to folate deficiency. In fact, folate supplementation is used clinically to alleviate these symptoms. Reduced PCFT-mediated intestinal folate absorption by methotrexate via competitive inhibition appears to be negligible based on investigations showing that folate absorption is unchanged in the presence of methotrexate [54]. Instead, folate deficiency in methotrexate-treated patients is likely due to the inhibition of folate metabolism that is necessary for DNA synthesis, or reduced reabsorption via PCFT in proximal tubules leading to increased renal elimination. Although methotrexate may not reduce folate absorption, other drugs such as the anti-inflammatory agent sulfasalazine have been identified as PCFT inhibitors at clinically relevant concentrations. Sulfasalazine is also associated with folate deficiency-related complications that are believed to result from reduced PCFT-mediated intestinal absorption of folate [55].

## 6. Disruption of Lactate Disposition

The disposition of several organic acids, including lactate, which promotes redox signaling and energy for oxidative metabolism, are mediated by MCT1-4 [16]. Lactate, a product of anaerobic glycolysis, is absorbed into enterocytes through MCT1, which is a 43 kDa protein that uses the symport of protons to drive cellular lactate uptake (Figure 1). MCT4 is expressed at the basolateral membrane of enterocytes and appears to be involved in lactate efflux into the portal vein [56]. MCT1 is expressed in almost all human tissues; thus, it plays a major role beyond enterocytes in mediating lactate uptake from plasma or facilitating efflux [16]. The genetic knockout of *Mct1* in mice is embryonically lethal, while *Slc16a1^−/+^* mice have neurodegenerative complications due to reduced lactate shuttling and decreased nutrient absorption [57]. Similarly, *MCT1*-inactive genetic variants in humans are associated with metabolic acidosis and diarrhea, although no changes in plasma lactate concentrations are observed [58].

MCT1 has become a drug target for multiple purposes. For example, gabapentin enacarbil, which is used clinically as an anticonvulsant (Table 2) [34,35], was designed as an MCT1 substrate to improve intestinal absorption and bioavailability. AZD3965 was recently designed as a MCT1 inhibitor (IC_50_ of 17 nM) with the goal of suppressing lactate uptake and altering glycolysis and pH in MCT1-overexpressing tumors [36,37]. The clinical utility of AZD3965 remains under development. The effects of MCT drug substrates or inhibitors on intestinal lactate absorption may require investigation considering that many have a higher affinity for MCTs than lactate (K_m_ < 3.5 mM). Indeed, metabolic acidosis has been reported following clinical exposure to AZD3965, along with increased urinary elimination of lactate and ketones [36]. These events occurred in the absence of lactate plasma concentration changes, consistent with *MCT1* genetic deficiency in humans. The increased urinary elimination of MCT1 substrates with AZD3965 is believed to result from a lack of MCT1-mediated reabsorption from proximal tubules, while a lack of changes in plasma may be due to compensation by MCT4, which is widely expressed among tissues (except ocular tissue) and is not inhibited by AZD3965. Despite compensatory mechanisms with plasma lactate concentrations and the complexity of factors that contribute to lactate metabolism and disposition, MCT1 inhibitors or substrates may still have significant potential to harm enterocyte homeostasis, especially considering that such compounds are commonly administered orally. Moreover, the genetically mediated loss of intestinal Mct1 activity alone in mice was shown to not only be sufficient in decreasing oral absorption of lactate but can alter microbiome contents, glucose homeostasis, and inflammation [59]. Overall, drugs with the potential to alter MCT1 activity are novel, and the potential harm or benefits of changes in MCT substrate disposition in humans taking inhibitors will require future investigation. These studies should also include establishing the role of MCTs in other tissues, including hepatocytes, or examining other transporters or enzymes involved in lactate disposition and metabolism that may compensate for MCT loss of function.

## 7. Disruption of Cholesterol Disposition

Cholesterol is a major structural component of human cell membranes and is a precursor for steroid hormone, bile acid, and vitamin synthesis. The absorption of cholesterol into enterocytes is mediated by the 145 kDa transporter NPC1L1 (Figure 1), followed by esterification and chylomicron secretion or efflux by the cholesterol efflux regulatory protein (CERP, encoded by the *ABCA1* gene) into the portal circulation [17]. NPC1L1 is also expressed at the apical membrane of hepatocytes. The importance of NPC1L1 is represented by genetically deficient mice, which have significantly reduced intestinal cholesterol absorption [60]. Similarly, human variations in the *SLC65A2* gene are associated with reduced intestinal absorption of cholesterol and reduced LDL concentrations [61].

The discovery of NPC1L1 provided clarity into the mechanism of action for ezetimibe, the first cholesterol absorption inhibitor approved to treat hypercholesterolemia. Specifically, ezetimibe acts by inhibiting NPC1L1 [62] and, to date, remains the only clinically used inhibitor of this transporter. Ezetimibe is effective in reducing cholesterol in patients either alone or in combination with a statin [63]. Accordingly, investigations into NPC1L1 inhibitors continue, often focusing on ezetimibe analogues. However, no compound superior to ezetimibe has been identified to date.

## 8. Pathological Alteration of Bile Acid Recirculation

**Figure 2:**
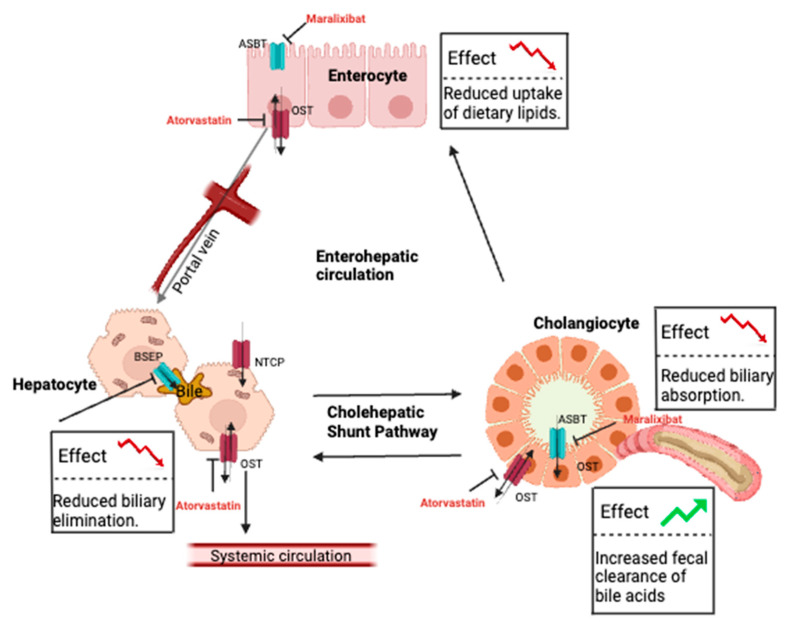
Interruption of bile acid recirculation. Major bile acid transporters involved in enterohepatic recirculation and cholehepatic shunt. Inhibition of ASBT (using maralixibat as an example) and OSTα/β (using atorvastatin as an example) in the gut lumen and cholangiocytes increases fecal clearance of bile acids. ASBT, apical sodium-dependent bile acid transporter. OSTα/β, organic solute transporter α/β. BSEP, bile salt export pump. NTCP, sodium–taurocholate cotransporting polypeptide. Created using BioRender.

Bile acids are steroidal compounds derived from cholesterol that are critical for the digestion of dietary lipids. These endogenous compounds are fundamental in the regulation of multiple metabolic processes, including cholesterol and insulin homeostasis [64]. Bile acid concentrations within the gastrointestinal tract are tightly regulated by the cholehepatic shunt and enterohepatic recirculation pathways to maintain digestive homeostasis (Figure 2) [18]. Bile acid uptake into enterocytes is mediated by ASBT, a 43 kDa protein located at the apical membrane that uses the symport of sodium to regulate transport activity. OSTα/β are facilitative transporters located at the basolateral membrane (with approximate molecular weights of 37 and 19 kDa, respectively) that move bile acids into the portal vein. OSTα/β are also expressed in hepatocytes, where they function with other transporters that include the uptake transporter sodium–taurocholate co-transporting polypeptide (NTCP, encoded by the *SLC10A1* gene) and OATPs, as well as the efflux transporters MRP1-4 and BSEP, to regulate bile acid concentrations. The importance of ASBT is supported by observations in rodent knockout models in which Asbt deficiency led to increased fecal cholesterol clearance, along with decreased bile acid pool and serum concentrations [65,66]. Genetic variation associated with reduced ASBT activity in humans is linked to primary bile acid malabsorption, which presents as congenital chologenic diarrhea and a loss of bile acid transport [67]. Genetic-mediated loss of OST function leads to cholestasis, liver fibrosis, and congenital diarrhea without changing systemic bile acid concentrations [68].

An increased clearance of bile acids may be beneficial for cholestatic disorders, including Alagille syndrome (ALGS), progressive familial intrahepatic cholestasis (PFIC), and biliary atresia. Regardless of cholestatic origin, these patients suffer from multiple adverse effects, including severe pruritis, which is associated with elevated serum bile acid concentrations. Targeting (inhibiting) intestinal bile acid transporters would decrease serum bile acid concentrations and is expected to reduce the severity of pruritis. Indeed, the inhibition of ASBT and OSTα/β alone can interrupt bile acid recycling and significantly increase the fecal clearance of bile acids [69,70].

ASBT inhibitors have been developed for cholestatic diseases in some countries. Maralixibat, approved in the United States in 2021, is indicated for the treatment of cholestatic pruritis in children with ALGS. The ICONIC trial showed that maralixibat lowered average observed itching scores by 2.3 points and serum bile acid concentrations by 36% by week 204 [40]. The IMAGINE-I and IMAGINE-II trials yielded similar results, and the ITCH trial showed a statistically significant improvement in observed itching scores by week 13 [41,42]. Currently, maralixibat is undergoing clinical trials as a treatment for pruritis in patients with PFIC types 1 and 2, biliary atresia, and generalized cholestatic liver disease (NCT02057718, NCT03905330, NCT04185363, NCT04524390, NCT04168385, and NCT04729751). Preliminary results indicate that this drug could be beneficial for these patient groups.

Odevixibat, another ASBT inhibitor approved in the United States and European Union in 2021, is indicated for the treatment of pruritis in children with PFIC types 1 and 2. The PEDFIC 1 trial showed statistically significant reductions in observed itching scores, scratching scores, and serum bile acid concentrations in patients receiving odevixibat for 22–24 weeks. A follow-up study, PEDFIC 2, confirmed these results. Patients also experienced improvements in sleep parameters in both PEDFIC trials [43]. Like maralixibat, odevixibat is currently undergoing clinical trials as a treatment for pruritis in patients with other cholestatic diseases [44].

A third ASBT inhibitor, elobixibat, is approved in Japan for the treatment of chronic idiopathic constipation due to the expected on-target effects of interrupting bile acid recycling and promoting bowel movements. In the United States, patients in the ACCESS trial who received ≥5 mg of the drug experienced at least a twofold increase in complete spontaneous bowel movements per week compared to a placebo (NCT01007123). Additionally, patients who received ≥10 mg of elobixibat experienced their first spontaneous bowel movement faster compared to placebo. As a class, the effectiveness of ASBT inhibitors in reducing serum bile acid concentrations render new therapeutic options for treating cholestatic and constipation-related disease states.

To date, there are no OSTα/β inhibitors approved for use in patients with cholestatic disease. Thus, evidence of the drug-induced disruption of OST substrate disposition is lacking. However, an in vitro study compared the effects of 77 test compounds on transporter activity in OSTα- and OSTβ-expressing cells (Flp-In 293) to that in mock cells (HEK293). Of these compounds, atorvastatin, ethinylestradiol, fidaxomicin, indomethacin, spironolactone, and troglitazone were strong OSTα/β inhibitors (≥50% inhibition relative to control) [45]. An in vitro study using fluorescence resonance energy transfer and OSTα/β-expressing cells identified clofazimine as another strong inhibitor [46]. Despite interest in developing OSTα/β inhibitors, a novel drug molecule has yet to be identified. This apparent lag in drug discovery may be due to the ASBT inhibitors showing promise as bile acid modulators or the potential negative impact on bile acid disposition in other OSTα/β-expressing cells such as hepatocytes. Additionally, unlike ASBT inhibitors, OSTα/β inhibitors must traverse the apical membranes of cholangiocytes and hepatocytes to reach the therapeutic (basolateral) target, creating an additional challenge with respect to drug design. To that end, both steroids and statins have shown OSTα/β inhibitory activity; thus, rigorous characterizations of those interactions may lead to the development of novel drug molecules as a new class of bile acid modulators.

## 9. Conclusions and Future Considerations

Considerable knowledge has been uncovered involving the disposition of nutrients, endogenous substrates, and drugs across the intestinal barrier, improving our understanding of the factors involved in intestinal absorption and relevant disease states. Through an abundance of in vitro, ex vivo, and in vivo animal models, along with clinical investigations, several transporters have been linked to the disposition of nutrients and endogenous substrates, including PCFT, NPC1L1, MCTs, SGLT1, THTR2, and ASBT. This insight has led to new clinical strategies or therapeutics to alleviate various disease symptoms, including those associated with cholestatic disease with new ASBT inhibitors.

Despite these advances in our understanding of the intestinal disposition of xeno- and endobiotics, further investigation is needed. The identification of unintended nutrient uptake transporter inhibitors such as fedratinib highlighted the potential patient risk of nutrient deficiencies, especially if chronic exposure is expected. Accordingly, the multiple potential transporter inhibitors described within this review will require follow-up investigations to assess their risk in promoting nutrient malabsorption. Such investigations should include in vitro and in vivo studies to assess the time dependency of inhibition or the time to transporter function recovery. Moreover, the unexpected trimethoprim-mediated increase in plasma thiamine concentrations highlights the need to consider transport or metabolic pathways within other tissues as compensatory mechanisms.

Future studies should expand beyond the examples provided and could consider innovative outcomes, such as the apparent changes in the gut microbiome content and colitis risk observed with diminished Pept1 activity in mice [71], as well as the risk of colitis in the absence of P-gp [72]. In silico approaches, which are commonly used to assess drug disposition changes under drug–drug or drug–nutrient interaction conditions, could also be devised to address the impact of these interactions on nutrient disposition and predict biological consequences. Finally, novel orphan intestinal transporters should be considered that may provide further insight into intestinal transporter-mediated drug absorption, interactions, and disease states, as well as new therapeutic strategies.

## Figures and Tables

**Table 1:** SGLT1, THTR2, PCFT, MCT1, NPC1L1, OST, and ASBT tissue mRNA expression, protein abundance, and membrane localization.

Transporter			Membrane Localization within Enterocytes		Measure of Expression *	Adipose Tissue	Adrenal Gland	Appendix	
SGLT1 (SLC5A1)			Apical [11]		mRNA [12]	Low	ND	Low	
					Protein [13]	ND	ND	ND	
THTR2 (SLC19A3)			Apical [14]		mRNA [12]	High	Low	Low	
					Protein [13]	ND	Low	Low	
PCFT (SLC46A1)			Apical [15]		mRNA [12]	Low	High	Low	
					Protein [13]	ND	Low	Low	
MCT1 (SLC16A1)			Apical [16]		mRNA [12]	Low	Medium	High	
					Protein [13]	ND	Medium	High	
NPC1L1 (SLC65A2)			Apical [17]		mRNA [12]	Low	Low	Low	
					Protein [13]	ND	ND	Medium	
OSTα (SLC51A)			Basolateral [18]		mRNA [12]	Low	Medium	Low	
					Protein [13]	ND	ND	ND	
OSTβ (SLC51B)			Basolateral [18]		mRNA [12]	Low	Low	Low	
					Protein [13]	ND	ND	High	
ASBT (SLC10A2)			Apical [18]		mRNA [12]	Low	Low	ND	
					Protein [13]	ND	ND	ND	
**Bone Marrow**	**Brain**	**Colon**	**Duodenum**	**Endometrium**	**Esophagus**	**Gall Bladder**	**Heart**	**Kidney**	**Liver**
Low	Low	Medium	High	Low	Low	High	High	Low	Low
ND	ND	ND	High	ND	ND	Low	ND	Medium	ND
Low	Low	Low	High	Low	Low	Medium	Low	Low	Medium
Low	Medium	Medium	Medium	Low	Medium	Medium	Medium	Medium	Medium
Low	Low	Low	High	Low	Low	Low	Low	Low	Low
ND	ND	Medium	Low	ND	ND	Low	Low	ND	ND
Low	High	High	High	High	Medium	High	High	Low	High
Low	Low	High	High	High	Medium	Medium	Medium	Medium	Medium
Low	Low	Low	High	Low	Low	Low	Low	Low	High
ND	ND	Low	High	ND	ND	Low	ND	Low	Medium
Medium	Low	High	High	Low	Low	Low	Low	Medium	High
ND	ND	ND	High	ND	ND	ND	ND	High	ND
ND	Low	High	High	Low	Low	Low	Low	Medium	Low
ND	ND	High	High	ND	ND	ND	ND	Medium	ND
ND	ND	Low	High	Low	ND	Low	ND	Medium	Low
ND	ND	ND	ND	ND	ND	ND	ND	ND	ND
**Lung**	**Lymph Node**	**Ovary**	**Pancreas**	**Placenta**	**Prostate Gland**	**Salivary Gland**	**Skin**	**Small Intestine**	
Low	Low	Low	Low	Low	Low	Low	Low	High	
ND	ND	ND	ND	ND	ND	ND	ND	High	
Low	Low	Low	Low	High	Low	Low	Low	Medium	
Low	ND	ND	Medium	High	ND	Medium	Low	Low	
Low	Low	Low	Low	Low	Medium	Medium	Low	High	
Low	ND	ND	Low	Low	ND	ND	ND	Low	
Low	Low	Low	Low	High	Medium	Low	Medium	High	
Medium	Medium	ND	ND	Medium	High	ND	Medium	Medium	
Low	Low	Low	Low	Low	Low	Low	Low	High	
ND	ND	ND	ND	ND	ND	ND	ND	High	
Low	Low	Low	Low	Low	Low	Low	Low	High	
ND	ND	ND	ND	ND	ND	ND	ND	High	
Low	Low	Low	ND	Low	Low	Low	Low	High	
ND	ND	ND	ND	ND	ND	ND	ND	High	
Low	Low	ND	ND	ND	ND	ND	Low	High	
ND	ND	ND	ND	ND	ND	ND	ND	High	
**Spleen**		**Stomach**		**Testis**	**Thyroid Gland**		**Urinary Bladder**		
Low		Low		Low	Low		Low		
ND		ND		ND	ND		ND		
Low		Low		Low	Low		Low		
ND		Medium		Medium	Medium		ND		
Medium		Low		Low	Low		Low		
ND		Low		High	Low		ND		
Low		High		High	Medium		Medium		
Low		High		High	ND		Low		
Low		Low		Low	Low		Low		
ND		ND		ND	ND		ND		
Low		Low		High	Low		Low		
ND		ND		ND	ND		ND		
Low		Low		Low	Low		Low		
ND		Medium		High	ND		ND		
ND		Low		ND	ND		ND		
ND		ND		ND	ND		ND		

ND, not detected. * mRNA: high indicates > 15 fragments per kilobase of exon per million mapped reads (FPKM), medium indicates 10–15 FPKM, and low indicates <10 FPKM. All mRNA data were previously published [12]. Protein: high, medium, and low based on immunohistochemistry staining intensity. Protein abundance data are reported from the human protein atlas [13].

**Table 2:** Nutrient- or endogenous-substrate-mediated transport with potential sensitivity to drug exposure.

Nutrient/Endogenous Substrate and Transport Process	Inhibitor (In Vitro IC_50_)	Level of Evidence	Reference(s)
Glucose uptake by SGLT1	Sotagliflozin (0.036 μM)—direct inhibitor	Reduced plasma glucose concentration in patients	[21,22]
	Erlotinib * (NA)—indirect inhibitor	Reduced glucose uptake in A549, MCF10A, H322, or H292 cells	[25,26,27]
	Lapatinib * (NA)—indirect inhibitor	Reduced glucose uptake in A549 or MCF10A2 cells	[26]
	Sorafenib * (NA)—indirect inhibitor	Reduced plasma glucose concentration in patients	[28]
	Dasatinib * (NA)—indirect inhibitor	Reduced plasma glucose concentration in patients	[28]
	Sunitinib * (NA)—indirect inhibitor	Reduced plasma glucose concentration in patients	[28]
	Imatinib * (NA)—indirect inhibitor	Reduced plasma glucose concentration in patients	[28]
Thiamine uptake by THTR2	Fedratinib (0.94–1.36 μM)—direct inhibitor	Onset of Wernicke’s encephalopathy in patients	
		Reduced thiamine uptake in Caco-2 and THTR2-overexpressing HEK293 cells	[29,30,31]
	Trimethoprim (5.6 μM)—direct inhibitor	Increased plasma thiamine concentration in patients	[32]
		Reduced thiamine uptake in Caco-2 and THTR2-overexpressing HEK293 cells	[30]
	Metformin (680 μM)—direct inhibitor	Reduced thiamine uptake in THTR2-overexpressing HEK293 cells	[31]
	Hydroxychloroquine (17 μM)—unknown if direct/indirect inhibitor	Reduced thiamine uptake in THTR2-overexpressing HEK293 cells	[31]
	Verapamil (141 μM)—unknown if direct/indirect inhibitor	Reduced thiamine uptake in THTR2-overexpressing HEK293 cells	[31]
Folate uptake by PCFT	Sulfasalazine (60 μM)—direct inhibitor	Reduced folate and methotrexate uptake in PCFT-overexpressing oocytes	[33]
Lactate uptake by MCT1	Phloretin (NA)—direct inhibitor	Reduced lactate uptake in MCT1-overexpressing oocytes	[16,34]
	Gabapentin enacarbil/XP-13512 (0.62 μM)—direct inhibitor	Reduced lactate uptake in Caco-2 cells and MCT1-overexpressing HEK293 cells and oocytes	[34,35]
	Quercetin (NA)—direct inhibitor	Reduced lactate uptake in MCT1-overexpressing oocytes	[16,34]
	AR-C155858 (NA)—direct inhibitor	Reduced lactate uptake in MCT1-overexpressing oocytes	[16]
	ADZ3965 (17 nM)—direct inhibitor	Metabolic acidosis risk; increased urinary elimination of lactate and ketone; no changes in lactate plasma concentrations in patients	[36,37]
Cholesterol uptake by NPC1L1	Ezetimibe (24 μM)—direct inhibitor	Reduced cholesterol uptake in NPC1L1-overexpressing MDCKII cells	[38]
		Reduced dietary cholesterol absorption in patients	[39]
Bile acid transport by ASBT	Maralixibat (0.3 nM)—direct inhibitor	Reduced serum bile acid concentrations in patients	[40,41,42]
	Odevixibat (0.10 nM)—direct inhibitor	Reduced serum bile acid concentrations in patients	[43,44]
	Elobixibat (0.53 nM)—direct inhibitor	Reduced complete spontaneous bowel movements per week in patients	NCT01007123
Bile acid transport by OSTα/β	Atorvastatin (NA)—unknown if direct/indirect inhibitor	Reduced dehydroepiandrosterone sulfate in OSTα/β-overexpressing HEK293 cells	[45]
	Ethinylestradiol (53 μM)—unknown if direct/indirect inhibitor	Reduced dehydroepiandrosterone sulfate in OSTα/β-overexpressing HEK293 cells	[45]
	Fidaxomicin (169 μM)—unknown if direct/indirect inhibitor	Reduced dehydroepiandrosterone sulfate in OSTα/β-overexpressing HEK293 cells	[45]
	Indomethacin (NA)—unknown if direct/indirect inhibitor	Reduced dehydroepiandrosterone sulfate in OSTα/β-overexpressing HEK293 cells	[45]
	Spironolactone (NA)—unknown if direct/indirect inhibitor	Reduced dehydroepiandrosterone sulfate in OSTα/β-overexpressing HEK293 cells	[45]
	Troglitazone (NA)—unknown if direct/indirect inhibitor	Reduced dehydroepiandrosterone sulfate in OSTα/β-overexpressing HEK293 cells	[45]
	Clofazimine (30–50 μM)—unknown if direct/indirect inhibitor	Reduced taurocholic acid transport across OSTα/β-overexpressing MDCK cells	[46]

* SGLT-mediated disposition was not measured specifically. IC_50_, concentration that inhibits 50% of transport activity. ASBT, apical sodium-dependent bile acid transporter. MCT1, monocarboxylic acid transporter 1. NPC1L1, Niemann-Pick C1-Like1 transporter. OSTα/β, organic solute transporter α/β. PCFT, proton-coupled folate transporter. SGLT1, sodium-glucose linked transporter 1. THTR2, thiamine transporter 2. NA, not available.

## Data Availability

All data are located within cited studies.
